# Exploring the mechanisms behind HIV drug resistance in sub-Saharan Africa: conceptual mapping of a complex adaptive system based on multi-disciplinary expert insights

**DOI:** 10.1186/s12889-022-12738-4

**Published:** 2022-03-07

**Authors:** Anneleen Kiekens, Bernadette Dierckx de Casterlé, Giampietro Pellizzer, Idda H. Mosha, Fausta Mosha, Tobias F. Rinke de Wit, Raphael Z. Sangeda, Alessio Surian, Nico Vandaele, Liesbet Vranken, Japhet Killewo, Michael Jordan, Anne-Mieke Vandamme

**Affiliations:** 1 Department of Microbiology, Immunology and Transplantation, Clinical and Epidemiological Virology, Institute for the Future, Rega Institute for Medical Research, KU Leuven, Leuven, Belgium; 2 Department of Public Health and Primary Care, Academic Centre for Nursing and Midwifery, KU Leuven, Leuven, Belgium; 3 Doctors With Africa CUAMM, Padua, Italy; 4 Department of Behavioural Sciences, Muhimbili University of Health and Allied Sciences, P.O Box 65015, Dar es Salaam, Tanzania; 5 Ministry of Health Community Development Gender Elderly and Children, Dar es Salaam, Tanzania; 6 Amsterdam Instiute for Global Health and Development (AIGHD), Amsterdam, the Netherlands; 7 Department of Pharmaceutical Microbiology, Muhimbili University of Health and Allied Sciences, P.O Box 65012, Dar es Salaam, Tanzania; 8 FISPPA Department, Università Degli Studi Di Padova, 35139 Padova, Italy; 9 Faculty of Economics and Business, Access To Medicine Research Center, KU Leuven, Leuven, Belgium; 10 Department of Earth and Environmental Sciences, Division of Bioeconomics, KU Leuven, Leuven, Belgium; 11 Department of Epidemiology and Biostatistics, Muhimbili University of Health and Allied Sciences, P.O Box 65001, Dar es Salaam, Tanzania; 12 Tufts University School of Medicine, Boston, USA; 13 Division of Geographic Medicine and Infectious Diseases, Tufts Medical Center, Boston, USA; 14 Tufts Center for Tufts Center for Integrated Management of Antimicrobial Resistance (CIMAR), Boston, USA; 15 Center for Global Health and Tropical Medicine, Unidade de Microbiologia, Instituto de Higiene E Medicina Tropical, Universidade Nova de Lisboa, Lisbon, Portugal

## Abstract

**Background:**

HIV drug resistance (HIVDR) continues to threaten the effectiveness of worldwide antiretroviral therapy (ART). Emergence and transmission of HIVDR are driven by several interconnected factors. Though much has been done to uncover factors influencing HIVDR, overall interconnectedness between these factors remains unclear and African policy makers encounter difficulties setting priorities combating HIVDR. By viewing HIVDR as a complex adaptive system, through the eyes of multi-disciplinary HIVDR experts, we aimed to make a first attempt to linking different influencing factors and gaining a deeper understanding of the complexity of the system.

**Methods:**

We designed a detailed systems map of factors influencing HIVDR based on semi-structured interviews with 15 international HIVDR experts from or with experience in sub-Saharan Africa, from different disciplinary backgrounds and affiliated with different types of institutions. The resulting detailed system map was conceptualized into three main HIVDR feedback loops and further strengthened with literature evidence.

**Results:**

Factors influencing HIVDR in sub-Saharan Africa and their interactions were sorted in five categories: biology, individual, social context, healthcare system and ‘overarching’. We identified three causal loops cross-cutting these layers, which relate to three interconnected subsystems of mechanisms influencing HIVDR. The ‘adherence motivation’ subsystem concerns the interplay of factors influencing people living with HIV to alternate between adherence and non-adherence. The ‘healthcare burden’ subsystem is a reinforcing loop leading to an increase in HIVDR at local population level. The ‘ART overreliance’ subsystem is a balancing feedback loop leading to complacency among program managers when there is overreliance on ART with a perceived low risk to drug resistance. The three subsystems are interconnected at different levels.

**Conclusions:**

Interconnectedness of the three subsystems underlines the need to act on the entire system of factors surrounding HIVDR in sub-Saharan Africa in order to target interventions and to prevent unwanted effects on other parts of the system. The three theories that emerged while studying HIVDR as a complex adaptive system form a starting point for further qualitative and quantitative investigation.

**Supplementary Information:**

The online version contains supplementary material available at 10.1186/s12889-022-12738-4.

## Background

### HIV drug resistance

HIV drug resistance (HIVDR) remains a threat to the effectiveness of antiretroviral therapy (ART). Over the last decade, major efforts have been made to achieve global 90-90-90 goals by 2020 and to end the HIV epidemic as a public health threat by 2030 [1]. However, levels of HIVDR are rising, compromising the effectiveness of ART and potentially also the efforts to attain the last 90 goal [2]. In 2017, mathematical modeling predicted that if left unchecked, excess levels of pretreatment HIVDR to the NNRTI drug class could directly lead to 890 000 AIDS deaths, 450 000 new infections, and 6.5 billion USD extra ART costs by 2030 in sub-Saharan Africa (SSA) [3]. Recently several cases of multi-drug class resistant HIV have been reported [4, 5].

Several causes of both pre-treatment HIVDR and acquired HIVDR have been described in the literature. Due to the high genetic variability of the virus, selective pressure stemming from a combination of incomplete adherence (defined here in the broader sense of not taking ART as prescribed, which can be influenced by a multitude of factors which are both within and out of the control of the clients themselves) and a low genetic barrier of ART may lead to the emergence of HIVDR [6]. In addition to biological and pharmacokinetic factors influencing the selection and emergence of HIVDR lie other, indirectly related factors. In a meta-analysis Shubber et al. identified diverse barriers to adherence such as forgetfulness, traveling, medication toxicity, stigmatization, food insecurity, alcohol or substance misuse [7]. Other crucial aspects to prevent HIVDR are for example sufficient ART availability and a well-functioning ART supply system [8]. These and other factors described in literature relate to several fields of science and in some cases also to other complex problems. For example, ART drugs have been reported to be used in a mixture of recreational drugs called whoonga in South Africa [9–11]. The complex problem of drug abuse, is therefore linked to HIVDR as this exposure to ART may have consequences for pre-treatment drug resistance.

Despite the fact that most of the factors contributing to HIVDR are presumed to be known, and that models to mitigate these causes have been built, pre-treatment HIVDR, especially in SSA, is still increasing [12].

### HIVDR as a complex adaptive system

As the factors influencing the emergence of HIVDR are numerous, have roots in different fields of science and are interconnected with other complex problems, we argue that HIVDR should be approached as a complex adaptive system (CAS), combining knowledge of diverse experts and stakeholders. Such systems have been defined by Plsek et al. as ‘a collection of individual agents with the freedom to act in ways that are not always totally predictable, and whose actions are interconnected so that one agent’s actions changes the context for other agents’ [13]. A successful intervention on one element of the system does not guarantee resolving the core problem. Rather, interventions should be planned keeping in mind the entire system, its particular dynamics and possible feedback loops and with the aim of reshaping the system in a favorable way [13, 14]. Feedback loops can be reinforcing or balancing, meaning that a change in a certain direction will either evolve into more change or balance itself out by propagating an opposite effect. CAS have been studied in several other contexts such as ecosystem management, healthcare management and obesity [15–17]. Moreover, the importance of using systems thinking in health care has been widely described in the literature [13, 14, 18–20]. In 2017, Rutter et al. described the need of approaching public health problems as complex systems in order to identify, implement and evaluate effective interventions [14]. Such interventions should be done at leverage points in the systems. These are points where a small intervention can have a large impact on the system [21]. Identifying leverage points is difficult and sometimes counterintuitive. Gaining insights in subsystems or feedback loops may therefore facilitate the identification of leverage points [22].

With this study, we aimed to make a first attempt at understanding the complexity behind HIVDR by combining the expertise and viewpoints from different HIVDR experts. In this article we describe how we identified three interconnected feedback loops influencing HIVDR by developing a systems map that represents the CAS of HIVDR in SSA based on the insights of international HIVDR experts from different disciplines. We discuss the insights gained from these feedback loops and possible applications for quantitative modelling, complexity-informed intervention design and policy development [23, 24].

## Methods

### Recruitment, inclusion criteria and setting

The systems map was designed based on semi-structured interviews with international experts from or with experience in SSA. For the purpose of this study, international experts were defined as stakeholders from diverse disciplines and institutions, working at an international level on HIVDR related to SSA and with a minimum of five years of experience. The participants were selected based on their expertise concerning HIVDR and with the aim of creating a mix of backgrounds and institutions covering all aspects of HIVDR. Purposive sampling was done starting from the expertise and connections of the Rega Institute and the Institute for the Future in Leuven, Belgium. This was supplemented with snowball sampling, using the expertise and connections of participants, and theoretical sampling, looking for the missing perspectives based on the emergent findings. They were contacted through email or in person when an opportunity presented itself, for example at international conferences. The interviews were held face to face (*n* = 6) or online over Skype or Zoom (*n* = 9) and were conducted in English. Semi-structured interviews of approximately 60 min were conducted until data saturation was reached, aiming to cover all possible factors influencing HIVDR in SSA. For the purpose of this study we describe data saturation as the point at which no new elements were uncovered in new interviews and no new connections which significantly changed the final conceptual model, were uncovered.

### Semi-structured interview guide

An interview guide was designed with the input of several HIVDR and social science experts and was adapted according to insights developed through analysis (Additional file 2). The guide contained three sections: the first section entailed sociodemographic questions concerning the interviewees gender, age and educational background. The questions of section two related to the interviewees professional and personal experience with HIV or HIVDR in SSA. The third and main section covered their perspectives on the factors influencing HIVDR. All experts were asked what, in their experience, were the main causes of HIVDR. As a general guideline, the interviewer aimed to cover the following four areas: causes related to 1) availability of ART at the healthcare centre, 2) PLHIV’s ability to fetch ART, 3) PLHIV taking ART as prescribed and 4) ART suppressing the viral load. Additionally, when causes outside these four areas came up, they were also further discussed. Subsequently, depending on the expertise of the participant, follow-up questions such as “What do you think is causing the situation you just mentioned?” aimed to clarify the deeper reasons behind some of those initially indicated causes.

### Data analysis

Analysis of the semi-structured interviews was inspired by the QUAGOL method and done simultaneously with the data collection [25]. After each interview a technical report was written describing relevant characteristics of the participant and interview context, helpful for understanding the data in their specific context. The interviews were transcribed verbatim by an external firm and the quality of each transcription was verified by listening to the audio tapes and correcting possible errors in the transcripts. Each transcript was (re-)read until a list of factors influencing HIVDR as well as connections between those factors, mentioned either explicitly or implicitly by the interviewee, was extracted. Connections were assigned a positive, negative or dual polarity. A positive connection indicates that the influencing and influenced element evolve both in the same direction (e.g. A—> B: when factor A increases, B increases too). A negative connection indicates that both elements evolve in the opposite direction (e.g. A- > B: when factor A increases, B decreases and vice versa). A dual connection indicates that both effects are possible. Take the following paraphrased quote as an example: “You know, sometimes people form peer support groups so that each month someone will pick up the medication for the whole group. This way people have to go only once every six months instead of on a monthly basis.” This would be translated into a negative arrow from “peer support group” to “required frequency of hospital visits”. Subsequently for each of the first six interviews separately, these factors were visualized in a small systems map while re-reading the interview again in order to visualize all the mentioned connections between these factors. Afterwards the separate systems maps of the first six interviews were merged together into one and from that point onwards data from the following interviews was added to the map. Throughout the analysis newly discovered insights were constantly compared with previous findings resulting in an iterative process of re-reading interviews and reviewing the detailed systems map.

The model was designed in Kumu, an online mapping tool which enables the user to save data such as interview quotes and memos for each element and connection [26]. In the first, confidential, version of the systems map, all interview quotes which mention a certain element or connection, are collected in the comment fields associated with the element or connection in the KUMU tool, facilitating our analysis. From this first draft systems map causal loops were identified manually as series of elements connected to each other in a circular way. Causal loops which contributed to the same mechanism were identified as a subsystem (this can be compared with a road map: all possible routs you could take to go from Brussels to Amsterdam would be classified together as the subsystem “routs from Brussels to Amsterdam”). Because the subsystems consisted of many elements and connections, they were conceptualized into one overall mechanism per subsystem which reflected the overall messages of interviews as well as possible. While each separate element and connection was mentioned in one or several interviews, the resulting feedback loops are based on the combination of knowledge from the different experts. The conceptualization of the subsystems was linked back to the original interviews, discussed with several stakeholders and strengthened with literature evidence.

## Results

### Systems map of factors influencing HIVDR as informed by the expertise of different HIVDR experts

In total 15 international experts were interviewed. Table 1 summarizes the scientific and institutional background of the interviewees. A diverse sample of experts with different expertise and institutional affiliation was reached, permitting us to gain insights in the various aspects of the CAS. Out of the 15 participants, 13 were researchers or had previous research experience in the field of HIVDR.

**Table 1:** Participant characteristics: different backgrounds and institution types of the interview participants. Note that some participants had a background in several fields of science or were working for more than one institution

Scientific background	N	Institution type	N
Medicine (public health/tropical medicine)	5	Global policy-making institution	3
Virology	4	Local policy-making institution	2
Epidemiology and public health	4	Hospital	2
Psychology	2	NGO	5
Finance	1	Pharmaceutical company	1
Human rights law	1	Insurance company	1
Engineering	1	University	3
Nursing science	1		
Economy	1		
Business	1		
Anthropology	1		

**Fig. 1:**
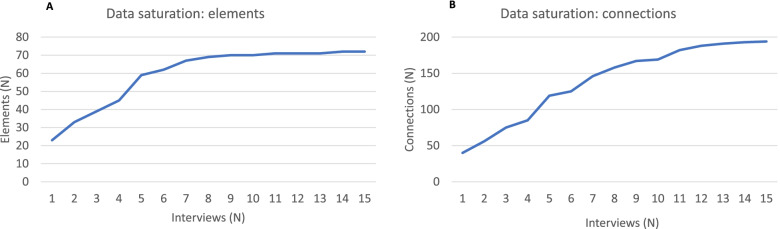
Data saturation curves. **A**) Number of elements in the systems map after each consecutive interview. **B**) Number of connections in the systems map after each consecutive interview

Data saturation for elements (factors influencing HIVDR) was reached after about nine interviews and for connections (pathways of influence between two elements) after 12 interviews (Fig. 1).

### The subsystems behind HIVDR

All elements and connections identified from the semi-structured interviews are represented in Table 2, Table 3 and Additional file 3. Based on this data, we visualized the system in two ways [27]. The first visualization divides the elements in five layers according to their relation to biology (elements and processes happening inside the body), individual factors (psychology, personal factors and behavior of adherence), social context (personal characteristics as a member of the community and baseline conditions in the community), healthcare system (treatment plan and healthcare organization), and ‘overarching’ factors (such as international policy, research and funding) (Additional file 1).

**Table 2:** Connections as presented in Fig. 2 and Additional file 1. The connection type represents the polarity of the connection. A positive connection type indicates that both elements evolve in the same direction (when element A increases, element B will increase too, and vice versa). A negative connection type indicates that both elements will evolve in the opposite direction (when element A increases, element B will decrease, and vice versa)

From	To	Type
Acceptance of HIV status	Adherence	+
Acceptance of HIV status	Engagement and retention in care	+
Acceptance of HIV status	Priority given to treatment	+
Acceptance of HIV status	HIV status disclosure	+
Accessibility of health centre (including safety)	Engagement and retention in care	+
Adherence	Drug levels in body	+
Adherence counselling	Understanding of HIV infection and treatment	+
Adherence counselling	Readiness to start taking ART	+
Administrative and political barriers	Individual and community empowerment	-
Administrative and political barriers	Timely acting on unsuppressed viral load	-
Administrative and political barriers	Well-functioning supply chain	-
ART treatment approach / policy	Timely acting on unsuppressed viral load	±
ART treatment approach / policy	Healthcare system workload	±
ART treatment approach / policy	Correct prescribing practices	±
ART treatment approach / policy	Required frequency of hospital visits	±
ART treatment approach / policy	Competence of healthcare workers	±
Assuring quality of ART	Efficiency of drug combination	+
Availability and quality of equipment	Timely acting on unsuppressed viral load	+
Availability of better drugs	Global effort to tackle HIVDR	-
Availability of better drugs	HIVDR selection	-
Community stigma and gossip	Engagement and retention in care	-
Community stigma and gossip	Distance to the healthcare centre	+
Community stigma and gossip	Self-stigmatisation	+
Community stigma and gossip	Healthcare provider stigma	+
Community stigma and gossip	Adherence	-
Community stigma and gossip	HIV status disclosure	-
Competence of healthcare workers	Timely acting on unsuppressed viral load	+
Competence of healthcare workers	Correct prescribing practices	+
Competence of healthcare workers	Adherence counselling	+
Competence of healthcare workers	Patient-provider relationship	+
Concerns about side effects of ART	Adherence	-
Concurrent disease and opportunistic infections	Feeling and looking ill	+
Concurrent disease and opportunistic infections	Pill burden	+
Concurrent disease and opportunistic infections	Drug-drug interactions	+
Concurrent disease and opportunistic infections	Healthcare system workload	+
Concurrent disease and opportunistic infections	Optimal absorption of drug	-
Correct prescribing practices	Efficiency of drug combination	+
Depression	Adherence	-
Depression	Priority given to treatment	-
Depression	Substance abuse	+
Distance to the healthcare centre	Accessibility of health centre (including safety)	-
Distance to the healthcare centre	Engagement and retention in care	±
Drug levels in body	Viral load suppression	+
Drug levels in body	Side effects of ART	+
Drug prices	Resource allocation with focus on population	-
Drug-drug interactions	Optimal absorption of drug	-
Efficiency of drug combination	Viral load suppression	+
Engagement and retention in care	Adherence	+
Engagement and retention in care	Financial situation	-
Engagement in alternative care	Engagement and retention in care	±
Engagement in alternative care	Optimal absorption of drug	-
Engagement in alternative care	Misinformation	±
Engagement in alternative care	Adherence	±
Engagement in risk behaviour	Transmission of HIV(DR)	+
Feeling and looking ill	Community stigma and gossip	+
Feeling and looking ill	Engagement and retention in care	±
Feeling and looking ill	Priority given to treatment	+
Feeling and looking ill	HIV status disclosure	+
Feeling and looking ill	Concerns about side effects of ART	+
Financial situation	Accessibility of health centre (including safety)	+
Financial situation	Timely acting on unsuppressed viral load	+
Financial situation	Migration	-
Financial situation	Food insecurity	-
Financial situation	Priority given to treatment	+
Food insecurity	Adherence	-
Food insecurity	Optimal absorption of drug	-
Forgetfulness	Adherence	-
Gender inequality	HIV status disclosure	-
Gender inequality	Adherence	-
Gender inequality	Engagement and retention in care	-
Gender inequality	Lower social status	+
Gender inequality	Engagement in risk behaviour	+
Global effort to tackle HIVDR	HIVDR Funding	+
Global effort to tackle HIVDR	ART treatment approach / policy	+
Having examples of well-functioning ART	Community stigma and gossip	-
Having examples of well-functioning ART	Acceptance of HIV status	+
Healthcare provider stigma	Engagement and retention in care	-
Healthcare provider stigma	Adherence counselling	-
Healthcare system workload	Adherence counselling	-
Healthcare system workload	Tracing of PLHIV	-
Healthcare system workload	Correct prescribing practices	-
Healthcare system workload	Timely acting on unsuppressed viral load	-
Healthcare system workload	Well-functioning supply chain	-
Healthcare system workload	Competence of healthcare workers	-
Healthcare system workload	Patient-provider relationship	-
Healthcare system workload	Job satisfaction and motivation of healthcare workers	-
HIV status disclosure	Social support	±
HIV status disclosure	Community stigma and gossip	+
HIV status disclosure	Engagement in risk behaviour	-
HIV status disclosure	Adherence	±
HIV status disclosure	Engagement and retention in care	+
HIVDR Funding	HIVDR Research focus	+
HIVDR Funding	Stock availability of ART and reagents	+
HIVDR Funding	Availability and quality of equipment	+
HIVDR Funding	Resource allocation with focus on population	±
HIVDR Funding	Need to show success of the ART programme	+
HIVDR Funding	Resistance (and subtype) testing	+
HIVDR Research focus	Availability of better drugs	+
HIVDR Research focus	ART treatment approach / policy	+
HIVDR Research focus	Required frequency of hospital visits	-
HIVDR Research focus	Resource allocation with focus on population	+
HIVDR selection	Global effort to tackle HIVDR	+
HIVDR selection	Viral load suppression	-
HIVDR selection	Transmission of HIV(DR)	+
HIVDR selection	Healthcare system workload	+
Hospital design	Community stigma and gossip	±
Hospital design	HIV status disclosure	±
Incentive to search for information	Understanding of HIV infection and treatment	+
Incentive to search for information	Misinformation	+
Individual and community empowerment	Timely acting on unsuppressed viral load	+
Individual education level	Understanding of HIV infection and treatment	+
Job satisfaction and motivation of healthcare workers	Well-functioning supply chain	+
Job satisfaction and motivation of healthcare workers	Timely acting on unsuppressed viral load	+
Linguistic issues	Adherence counselling	-
Lower social status	Engagement and retention in care	-
Lower social status	Community stigma and gossip	+
Lower social status	Healthcare provider stigma	+
Migration	Healthcare system workload	+
Migration	Well-functioning supply chain	-
Migration	Engagement and retention in care	-
Misinformation	Understanding of HIV infection and treatment	-
Misinformation	Community stigma and gossip	+
Misinformation	Engagement in alternative care	+
Misinformation	Engagement in risk behaviour	+
Need to show success of the ART programme	HIVDR Funding	+
Need to show success of the ART programme	Administrative and political barriers	+
Optimal absorption of drug	Drug levels in body	+
Patient-provider relationship	Understanding of HIV infection and treatment	+
Patient-provider relationship	Engagement and retention in care	+
Patient-provider relationship	Adherence counselling	+
Patient-provider relationship	HIV status disclosure	+
Peer support group	Required frequency of hospital visits	-
Peer support group	Understanding of HIV infection and treatment	+
Pill burden	Pill fatigue	+
Pill burden	Side effects of ART	+
Pill fatigue	Adherence	-
Priority given to treatment	Adherence	+
Priority given to treatment	Engagement and retention in care	+
Punitive laws for MSM and sex workers	Engagement and retention in care	-
Punitive laws for MSM and sex workers	Transmission of HIV(DR)	+
Punitive laws for MSM and sex workers	Community stigma and gossip	+
Punitive laws for MSM and sex workers	ART treatment approach / policy	-
Quality of data systems	Tracing of PLHIV	+
Quality of data systems	Well-functioning supply chain	+
Quality of data systems	Timely acting on unsuppressed viral load	+
Readiness to start taking ART	Adherence	+
Religious beliefs	Self-stigmatisation	+
Religious beliefs	Engagement in alternative care	+
Required frequency of hospital visits	Engagement and retention in care	-
Required frequency of hospital visits	Healthcare system workload	+
Resistance (and subtype) testing	Correct prescribing practices	+
Resource allocation with focus on population	ART treatment approach / policy	+
Resource allocation with focus on population	Adherence	+
Self-stigmatisation	Acceptance of HIV status	-
Self-stigmatisation	HIV status disclosure	-
Self-stigmatisation	Depression	+
Side effects of ART	Feeling and looking ill	+
Side effects of ART	Adherence	-
Side effects of ART	HIV status disclosure	+
Social obligations	Financial situation	-
Social obligations	Priority given to treatment	-
Social support	Adherence	+
Stock availability of ART and reagents	ART treatment approach / policy	+
Stock availability of ART and reagents	Timely acting on unsuppressed viral load	+
Stock availability of ART and reagents	Job satisfaction and motivation of healthcare workers	+
Stock availability of ART and reagents	Required frequency of hospital visits	-
Stock availability of ART and reagents	Adherence	+
Substance abuse	Forgetfulness	+
Timely acting on unsuppressed viral load	Efficiency of drug combination	+
Tracing of PLHIV	Engagement and retention in care	+
Tracing of PLHIV	Timely acting on unsuppressed viral load	+
Transmission of HIV(DR)	Efficiency of drug combination	-
Transmission of HIV(DR)	Healthcare system workload	+
Understanding of HIV infection and treatment	Self-stigmatisation	-
Understanding of HIV infection and treatment	Engagement in risk behaviour	-
Understanding of HIV infection and treatment	Incentive to search for information	-
Understanding of HIV infection and treatment	Engagement and retention in care	+
Understanding of HIV infection and treatment	Adherence	+
Understanding of HIV infection and treatment	Acceptance of HIV status	+
Understanding of HIV infection and treatment	Individual and community empowerment	+
Understanding of HIV infection and treatment	Priority given to treatment	+
Understanding of HIV infection and treatment	Community stigma and gossip	-
Understanding of HIV infection and treatment	Engagement in alternative care	-
Viral load suppression	HIVDR selection	-
Viral load suppression	Concurrent disease and opportunistic infections	-
Viral load suppression	Required frequency of hospital visits	-
Viral load suppression	Healthcare system workload	-
Viral load suppression	Transmission of HIV(DR)	-
War and disease outbreaks	Accessibility of health centre (including safety)	-
War and disease outbreaks	Timely acting on unsuppressed viral load	-
War and disease outbreaks	Well-functioning supply chain	-
War and disease outbreaks	Migration	+
Well-functioning supply chain	Peer support group	+
Well-functioning supply chain	Stock availability of ART and reagents	+

**Table 3:** Overview of elements included in each factor of Fig. 2

Adherence motivation subsystem		Healthcare system burden		ART overreliance subsystem	Interconnected wicked problems
**Psychosocial**	Social support	**Quality of care**	Timely acting on unsuppressed viral load	**Global effort to tackle HIVDR**	Food insecurity
Acceptance of HIV status	Substance abuse	Accessibility of health centre (including safety)	Tracing of PLHIV	Drug prices	Gender inequality
Community stigma and gossip	**Adherence and retention**	Adherence counselling	Well-functioning supply chain	Global effort to tackle HIVDR	Lower social status
Concerns about side effects of ART	Adherence	Administrative and political barriers	**Health literacy and empowerment**	HIVDR funding	Migration
Depression	Engagement and retention in care	ART treatment approach / policy	Individual and community empowerment	Need to show success of the ART programme	Punitive laws for MSM and sex workers
Engagement in risk behaviour	Engagement in alternative care	Assuring quality of ART	Individual education level	Research focus	War and disease outbreaks
Financial situation	**Clinical manifestations**	Availability and quality of equipment	Incentive to search for information	Resource allocation with focus on population	
Forgetfulness	Concurrent disease and opportunistic infections	Patient-provider relationship	Misinformation	**Availability of ART with a higher genetic barrier**	
Having examples of well-functioning ART	Feeling and looking healthy	Competence of healthcare workers	Religious beliefs	Availability of better drugs	
HIV status disclosure	Side effects of ART	Correct prescribing practices	Understanding of HIV infection and treatment		
Hospital design	**Biology**	Distance to the healthcare centre	**Health system resources**		
Linguistic issues	Drug levels in body	Healthcare provider stigma	Healthcare system workload		
Pill burden	Drug-drug interactions	Job satisfaction and motivation of healthcare workers			
Pill fatigue	Efficiency of drug combination	Peer support group			
Priority given to treatment	HIVDR selection	Quality of data systems			
Readiness to start taking ART	Optimal absorption of drug	Required frequency of hospital visits			
Self-stigmatisation	Transmission of HIV(DR)	Resistance (and subtype) testing			
Social obligations	VL suppression	Stock availability of ART and reagents			

**Fig. 2:**
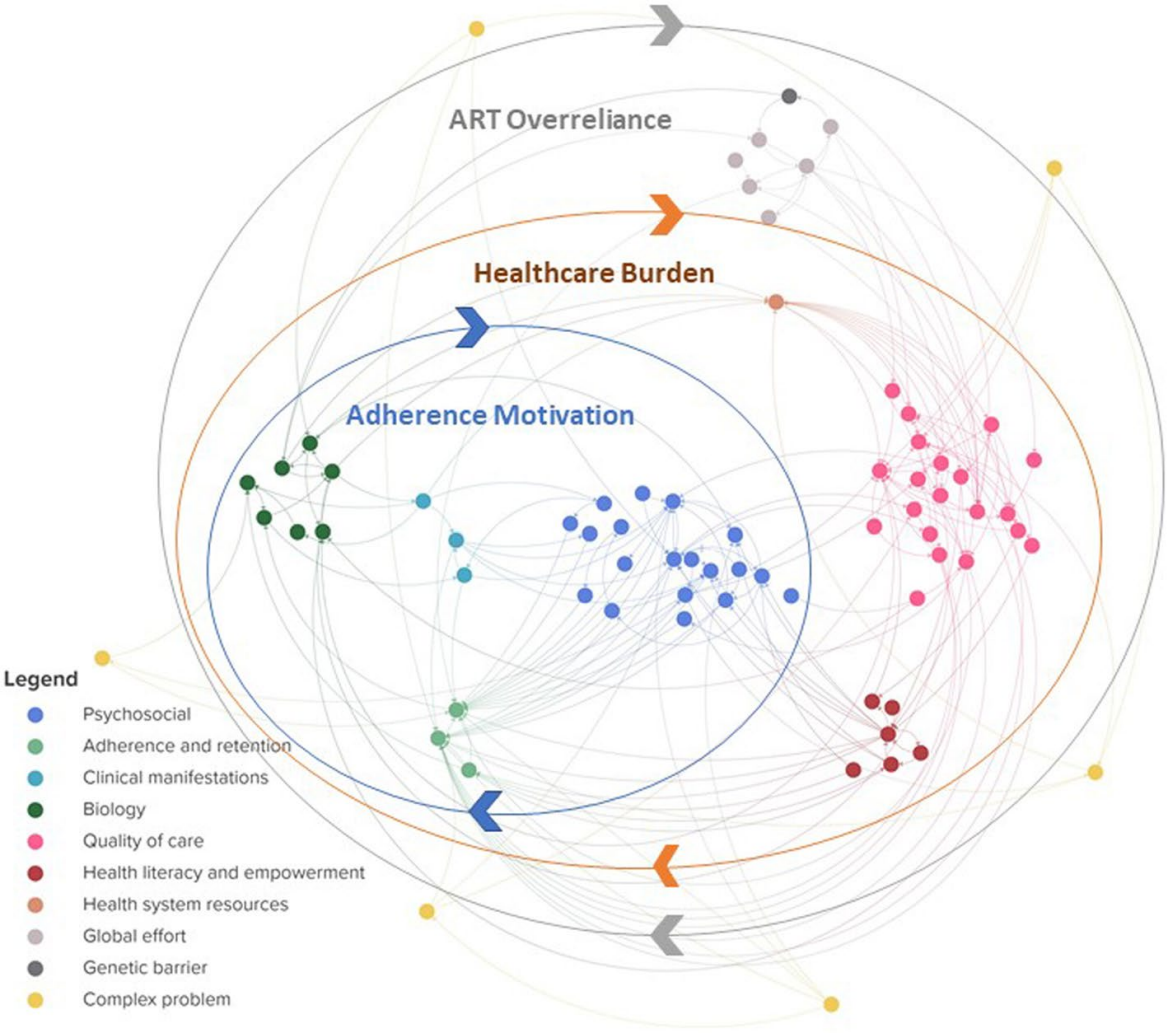
Clustered systems map visualizing three interconnected subsystems. Each cluster of elements is represented in a different colour, corresponding to the colours used in Fig. 3 and connects elements related to a certain theme. Note that all elements and connections represented here are the same as the ones presented in Additional file 1 but organized in clusters instead of in layers. Three main subsystems are indicated in the blue, orange and grey overlaying circles. An interactive overview this map, can be found in Additional file 3 ([27], page 1])

For the second visualization we grouped the same elements and connections in ten different thematic clusters (Fig. 2) ([27], page 1). The clusters represent elements belonging to the same themes identified in the interview data, being adherence and retention in care, biology, clinical manifestations, complex problems, genetic barrier of the medication, global effort to tackle HIVDR, health literacy and empowerment, health system resources, psychosocial factors and quality of care. The elements included in each cluster are presented in Table 3. When visualizing these clusters and the connections between them, three major feedback loops or sub-systems emerge, indicated by the three circles in Fig. 2.

#### Adherence motivation subsystem

The first subsystem suggests a mechanism at the personal level through which people living with HIV (PLHIV) may alternate between periods of optimal and suboptimal adherence. In different periods of their lives, PLHIV may give more or less priority to their treatment depending on several factors. When less priority is given to the ART and doses are missed, the viral load will not be suppressed and HIV related illness may develop. When feeling physically unwell, treatment may again be prioritized over other activities leading to a better adherence. When the viral load is suppressed and the individual feels better, other activities may take precedent and doses of ART may be skipped. When studying this subsystem, it is important to keep in mind that this alternating behavior can occur only a limited number of times before HIVDR emerges, after which optimal adherence will not lead to a better physical condition anymore.

We also note that not all individuals follow the pathways of this subsystem. PLHIV may fail to adhere even when feeling physically ill, or on the contrary, may have a continuous optimal adherence. This interplay between factors influencing an individual’s adherence has recently been described in a qualitative systematic review [28]. The authors describe how a combination of factors can lead to the decision of PLHIV to either adhere to ART or not and how this is a dynamic process of switching between adherence and non-adherence.

#### Healthcare burden subsystem

The second subsystem is situated at the programme level and relates to the burden on the healthcare system which, when too high, may jeopardize the quality of service delivery. Services provided at the healthcare center, such as adherence counseling, viral load testing or pill pick-up are essential to sustain viral load suppression but may be compromised when the healthcare system is overburdened. This may lead to delayed acting on a detectable viral load which on its turn leads to emergence of HIVDR and/or transmission of HIV(DR), requiring additional counseling and viral load tests. This, on its turn, increases the healthcare system workload. In short, this loop represents a sequence of events through which a high burden on the healthcare system amplifies itself. On the programme level, a high burden on the healthcare system may lead to delays in acting on non-suppressed viral load as the testing itself may be delayed due to insufficient laboratory and sample transport capacity or the healthcare workers may not have time to file reports or to return test results. HIVDR emergence resulting from a delay in acting on unsuppressed viral load in turn contributes to an increase in overall HIVDR burden at the personal and programme level. The World Health Organization reports that, though the African region carries the highest disease burden, they have the highest population/provider ratios [29]. In line with our findings, a study in Cameroon identified high health system workload as a possible risk factor for emerging HIVDR [30].

#### ART overreliance subsystem

At the population level, the availability of ART with a high potency and a high genetic barrier for resistance such as combinations including second generation integrase inhibitors offers a new and promising line of therapy. However, several interviewees expressed the concern that resistance against second generation integrase inhibitors such as Dolutegravir will eventually arise given that the first cases of resistance have already been reported [31–33]. With the introduction of integrase inhibitor-based ART in SSA, highly active treatment with a low risk to emergence of drug resistance, policy makers and in particular doctors, risk to overly rely on the effectiveness of the treatment. This shifts the healthcare focus to increasing the numbers of PLHIV on treatment at the cost of assuring high quality care for all. However, when adherence issues are left unsolved, the possibility of developing resistance against new ART regimens, despite their high genetic barrier, remains. This finding is supported by the review of Hamers et al. and by the findings of the ADVANCE trial that pre-treatment HIVDR to NRTIs and/or NNRTIs predicts virologic failure for regimens containing Dolutegravir [34, 35]. Altogether, this subsystem suggests that the use of ART with a higher genetic barrier to resistance alone may not be sufficient to prevent HIVDR and should always be supported by high quality service delivery. We currently see an interest in long-acting drugs with a high genetic barrier to drug resistance, which may facilitate adherence, but may again result in overconfidence, thereby increasing the risk of HIVDR in the long run if not implemented in the context of a systems approach. A similar reasoning has been made by Inzaule et al., who point out the challenges associated with the roll-out of dolutegravir such as reduced effectiveness of the therapy due to NRTI resistance and uncertainty about dolutegravir resistance due to insufficient access to viral load testing [36].

**Fig. 3:**
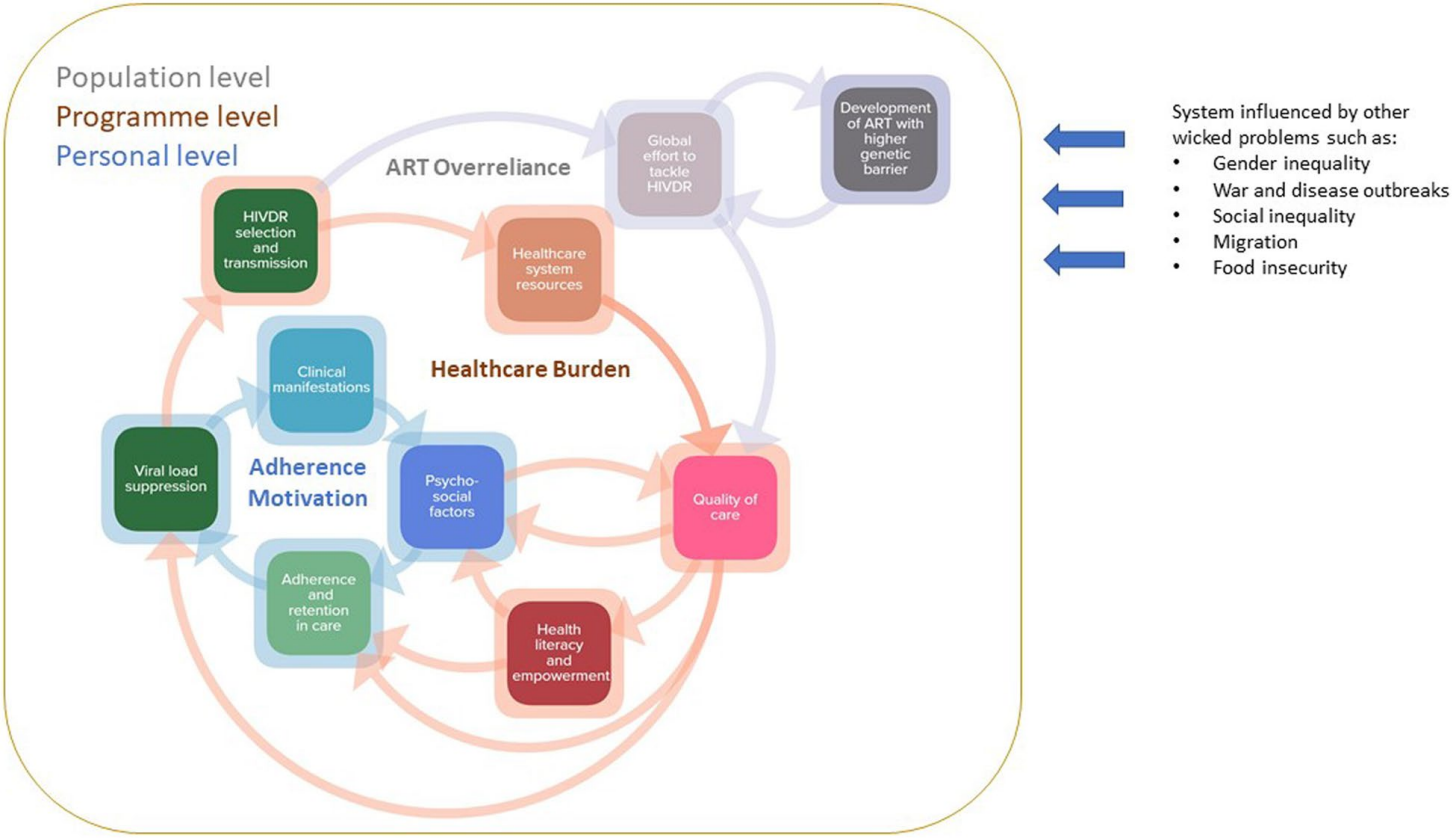
Three identified interconnected subsystems driving HIVDR. The adherence motivation subsystem at the personal level, the healthcare burden subsystem at programme level and the ART overreliance subsystem at the population level. Each square in this map represents a cluster of Fig. 2, indicated by the corresponding colours.

When interpreting the subsystems described above it is important to keep in mind that they are constantly influenced by each other and by other complex problems such as food insecurity, gender inequality or war and disease outbreaks. Figure 3 represents a summarized version of the three subsystems as presented in Fig. 2.

### Subsystem interactions

The three subsystems described above exist on different societal levels (personal, programme and population level) and are intrinsically linked with each other. The alternating adherence subsystem takes place on the personal level until HIVDR emerges, at which point the individual will add to the burden of the healthcare system. The increased burden on the healthcare system may then impact the overall quality of care, which in turn may impact the adherence of PLHIV through a delayed switch in ART after detection of viral non-suppression, thus increasing the chances of personal- and population-level HIVDR emergence. Diminished quality of ART service delivery may also impact adherence counselling and support, thereby directly impacting the alternating adherence subsystem at the personal level. Both pathways will eventually lead to an increase in HIVDR, which is reacted upon at the population level by researching and developing new drugs that are more forgiving with respect to adherence (e.g. long-acting drugs) and that have higher genetic barriers to resistance. Policy makers overly relying on these new ART regimens may shift focus away from high quality service delivery and HIVDR prevention measures. As described above, decreased quality of care may then impact the healthcare system burden at the population level and/or alter personal-level adherence.

The HIVDR system is influenced by several other complex problems at different points in the three subsystems. Food insecurity for example, may negatively affect adherence considering PLHIV have to take the ART with a meal each day. Other examples are political instability and disease outbreaks (such as the COVID-19 pandemic), which may destabilize the healthcare system, increase the burden on healthcare personnel and may cause PLHIV to have priorities other than adherence to ART.

## Discussion

In this paper we approached HIVDR, by our knowledge for the first time as a CAS by combining the perspectives of experts from diverse disciplines. We visualized the CAS of factors influencing HIVDR in two ways: a layered and a clustered view. We then summarized this detailed systems map into three interconnected subsystems influencing HIVDR emergence. We want to highlight that other ways of summarizing the detailed systems map are possible, but the three subsystems presented here were identified by the researchers as the most prominent ones throughout a process of analysis and stakeholder feedback.

The designed systems map provides insight in some properties of CASs such as emergence, adaptation and feedback and allowed to visualize the three interconnected subsystems [37]. The interplay between factors influencing adherence is an example of emergence, which indicates a phenomenon that cannot be predicted purely based on the elements related to it but which rather emerges from a complex interplay between the factors. Adherence is influenced by factors stemming from each of the five layers and is influenced at both personal, programme and population level. Whether PLHIV adhere to treatment or not depends on the interplay between those surrounding factors which are constantly changing over time. Adaptation describes how interventions in the system can lead to behavioral changes. Our systems map shows that the implementation of second-generation integrase inhibitors could lead to a change in adherence as a result of the overreliance of policy makers and doctors and depending on how the new therapy is introduced to the community and whether education and other support is provided. The feedback loops summarized here in the three subsystems reveal the interconnectedness between subsystems at different population levels and between factors of different layers and disciplines. This also underlines the need to reflect on the entire system surrounding HIVDR when planning an intervention.

An important shortcoming of this study is that only expert viewpoints were included. To make up for this, we aimed to include experts who have close contact with PLHIV and thus have insights in their perspectives. However, in order to design locally tailored interventions, the systems maps should be strengthened with insights from PLHIV and local actors. In follow-up work that has in the meantime been published, the adapted systems map based on the perspectives of local actors and PLHIV provided us with a better understanding of the personal and context dependent factors such as stigmatization or food insecurity [38]. This shifted the focus of the map with perspectives of local actors and PLHIV towards the “adherence motivation loop”, compared to the work presented here. For other study sites, perspectives of PLHIV and other local stakeholders such as local doctors or politicians, religious leaders, and other people of local influence could also help us better understand the differences in perspectives between those groups and identify possible gaps between science and practice. We also need to acknowledge that the mapping was done based on facts but also viewpoints and experiences of international experts. Combining the expertise of multidisciplinary HIVDR experts in a systems map has allowed us to identify the three potentially interesting theories, represented by the three subsystems above, which may not have surfaced through disciplinary or purely quantitative research. This qualitative approach was important to deepen our understanding of the CAS, before future quantitative efforts on specific parts of the system can be done [39].

### Applications

Our study illustrates the added value of qualitative methodology to visualize the complexity and dynamics of a system. This may help decision makers to gain insight into the systems complexity and to identify leverage points in order to design targeted and complexity-informed interventions. This methodology can be transferred to study HIVDR in specific settings or could be used to gain insights into other complex problems. Moreover, the content of the model presented in this study may (partially) be extrapolated to other chronic diseases such as diabetes or obesity in order to understand their drivers and feedback loops.

The conceptual model presented here also lays the basis for quantitative mathematical modelling of the factors influencing HIVDR. This will allow quantitative modelers to collect data on relevant parameters in the system to monitor any changes, desired or not, in the entire system. An important advantage of basing a quantitative model on this conceptual map lies in the multidisciplinary manner this map was developed, therefore identifying mechanisms which might not have been identified using a monodisciplinary approach.

## Conclusion

We successfully visualized the CAS surrounding HIVDR which is influenced by a complex and interconnected system of factors, transcending disciplines and population levels. This allows us for the first time to study the emergent and adaptive properties of the CAS and to distinguish feedback loops. The model suggests that i) overreliance on ART with a low risk to HIVDR emergence may be a driver for future HIVDR against those same ART; ii) when exceeding a certain threshold, the burden on the healthcare system amplifies itself; and iii) adherence tends to vary given that it is very individual- and context-dependent and might therefore be difficult to influence directly. A deeper understanding of the different aspects of this system will help decision makers to identify leverage points in order to design targeted and effective interventions in line with the complexity of the system.

## Supplementary Information

**Additional file 1.** HIVDR as a CAS, visualized in layers. Each element represents a factor influencing HIVDR and each line represents a connection between two factors. Factors are organized in five layers according to their connection with biology, the individual, the social context, the healthcare system and ‘overarching’. A detailed and interactive version of this map is included in Additional file 1 ([27], page 2).

**Additional file 2.** Interview guide.

**Additional file 3.** Interactive systems map and data set Excel file. Additional file 3 contains a weblink to an online interactive version of the systems maps included in the manuscript as Fig. 2 and Additional file 1. The second and third tab contain the data on the elements, connections, descriptions, connection type, layer category, subsystem category and cluster category necessary to recreate the interactive systems maps in Kumu. Moreover, the code for recreating both the layered view and the subsystem view are provided on tab 4 and 5.

## Data Availability

All data generated or analyzed during this study are included in this published article and its supplementary information files.

## Abbreviations

ART: Antiretroviral therapy
CAS: Complex adaptive system
HIVDR: HIV drug resistance
PLHIV: People living with HIV
SSA: sub-Saharan Africa
