# Supplementary figures and images for: Exploring the mechanisms behind HIV drug resistance in sub-Saharan Africa: conceptual mapping of a complex adaptive system based on multi-disciplinary expert insights
Source: BMC Public Health. 2022 Mar 7;22:455. doi: 10.1186/s12889-022-12738-4 (PMC8899794; doi:10.1186/s12889-022-12738-4)

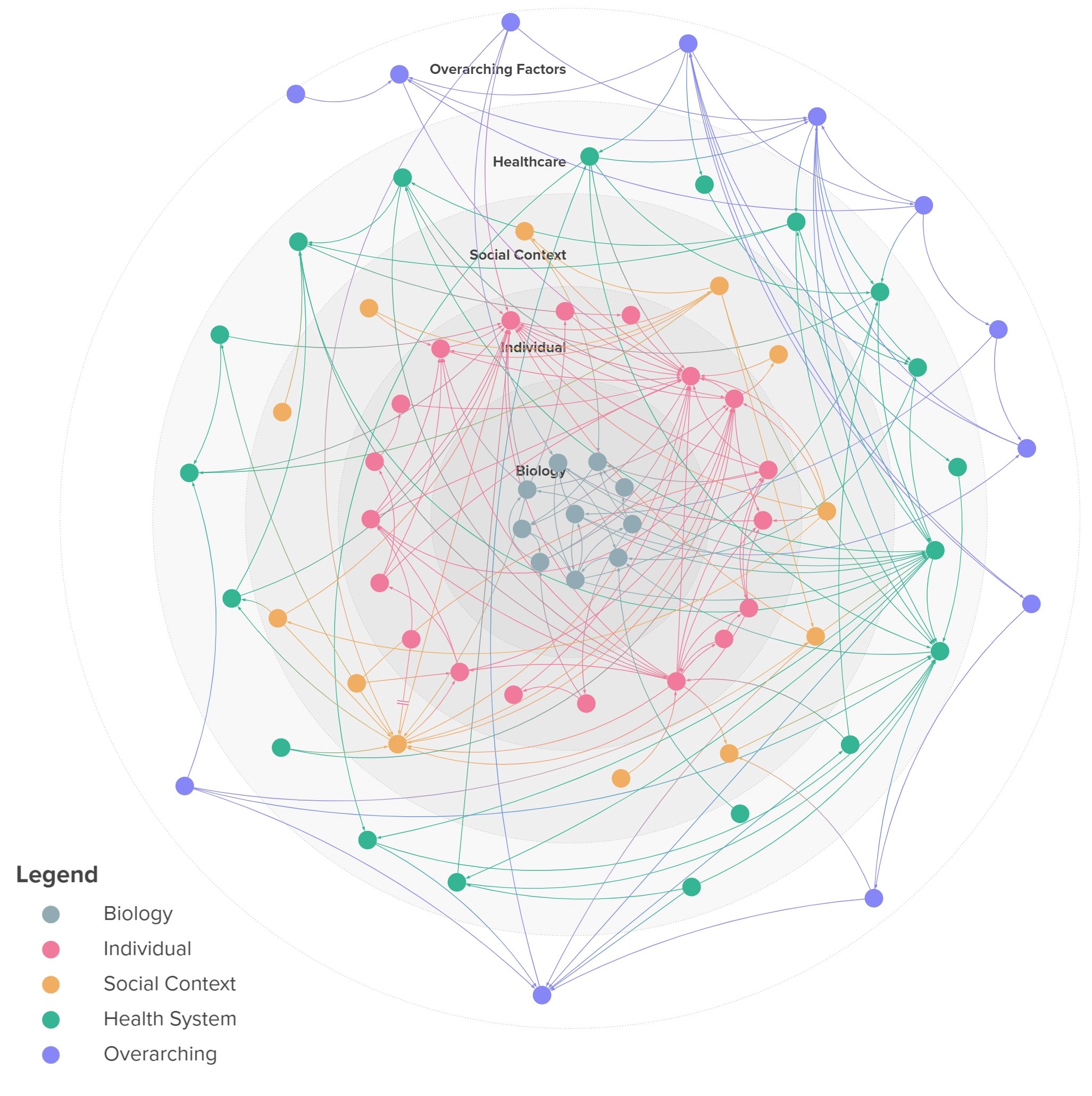

Supplement: Supplementary file 1 — Additional file 1. HIVDR as a CAS, visualized in layers. Each element represents a factor influencing HIVDR and each line represents a connection between two factors. Factors are organized in five layers according to their connection with biology, the individual, the social context, the healthcare system and ‘overarching’. A detailed and interactive version of this map is included in Additional file 1 ([27], page 2). [file 12889_2022_12738_MOESM1_ESM.jfif]
